# Supplementary material for: Immunization as Protection Against Long COVID in the Americas: A Scoping Review
Source: Vaccines (Basel). 2025 Jul 31;13(8):822. doi: 10.3390/vaccines13080822 (PMC12390208; doi:10.3390/vaccines13080822)

vaccines

MDPI

Review

Immunization as protection against long COVID in the Americas: A scoping review

Gabriela, Zambrano-Sánchez, MD, MSc [1,2], Josue, Rivadeneira MD, MSc [1,3,4], Carlos, Manterola MD, PhD [4,5], Tamara, Otzen PhD [*4,5], Luis, Fuenmayor-González MD and MSc [*1,3]

1 Universidad Central del Ecuador, Facultad de Ciencias Médicas, Unidad de Revisiones Sistemáticas y Metaanálisis-URMA, Quito-Ecuador, 170403
2 Hospital de Especialidades Eugenio Espejo, Quito-Ecuador, 170403
3 Zero Biomedical Research, Quito-Ecuador, 170103
4 Universidad de La Frontera, Doctorado en Ciencias Médicas, Temuco-Chile, 01145
5 Núcleo Milenio de Sociomedicina, Santiago-Chile, 01145
* Correspondence: correspondence to LF-G : lefuenmayor@uce.edu.ec; TO: tamara.otzen@ufrontera.cl

Supplementary

Table S1. Main concepts and related keywords

Concept	Keywords	MeSH term	Emtree term
Long COVID	Long COVID, Long COVID-19, Long COVID syndrome, Post COVID, Post COVID-19, Post COVID syndrome, Post-acute COVID-19 syndrome, Post Acute COVID 19 Syndrome, Post-Acute COVID-19 Syndromes, Post-Acute Sequelae of SARS-CoV-2 Infection, Post Acute Sequelae of SARS CoV 2 Infection, Post-COVID Conditions, Post-COVID Condition, Post COVID Conditions, PASC Post Acute Sequelae of COVID-19, Post-Acute Sequelae of COVID-19, COVID-19 Post-Acute Sequelae, Long COVID Condition, Post COVID condition, Long hauler COVID, COVID long-hauler, Long haul COVID	"Post-Acute COVID-19 Syndrome" [MeSH]	'long COVID'/exp
Immunization	SARS2 Vaccine*; Vaccine*, SARS2; Coronavirus Disease 2019 Vaccine*; Coronavirus Disease 2019 Virus Vaccine*; Coronavirus Disease-19 Vaccines; Coronavirus Disease 19 Vaccines<br>Vaccines, Coronavirus Disease-19; Coronavirus Disease-19 Vaccine; Coronavirus Disease 19 Vaccine; Vaccine, Coronavirus Disease-19<br>COVID 19 Vaccine*; Vaccine*, COVID 19<br>2019-nCoV Vaccine; 2019 nCoV Vaccine	"COVID-19 Vaccines"[MeSH]	'SARS-CoV-2 vaccine'/exp

	Vaccine*, 2019-nCoV; 2019 Novel Coronavirus Vaccines; SARS-CoV-2 Vaccine; SARS CoV 2 Vaccine; Vaccine*, SARS-CoV-2; 2019-nCoV Vaccines; 2019 nCoV Vaccines; COVID-19 Vaccine; Vaccine, COVID-19; SARS Coronavirus 2 Vaccines; COVID-19 Virus Vaccines; COVID 19 Virus Vaccines; Virus Vaccines, COVID-19; COVID-19 Virus Vaccine; COVID 19 Virus Vaccine; Vaccine*, COVID-19 Virus; Virus Vaccine, COVID-19; COVID19 Virus Vaccines; Virus Vaccines, COVID19; COVID19 Virus Vaccine; Vaccine*, COVID19 Virus; Virus Vaccine, COVID19 COVID19 Vaccine*; Vaccine*, COVID19; SARS-CoV-2 Vaccines; SARS CoV 2 Vaccines; 2019 Novel Coronavirus Vaccine		
Efficacy	Efficacy, Vaccine; Vaccine Effectiveness, Vaccine Efficacy, Effectiveness	"Vaccine Efficacy" [MeSH]	'vaccine'/exp 'comparative effectiveness'/exp 'drug efficacy'/exp
Severity	Severity; Illness Index Severities; Illness Index Severity	"Severity of Illness Index" [MeSH]	'COVID-19 Severity score'/exp 'disease severity'/exp
Prevention	prevention and control; preventive measures preventive therapy; prophylaxis; control; prevention; Prevention, Secondary; Preventions, Secondary; Secondary Preventions; Disease Prevention, Secondary	"prevention and control"[Subheading] "Secondary Prevention"[Mesh]	'prevention'/exp 'prophylaxis'/exp 'infection prevention'/exp 'prevention and control'/exp
Location	Latin America; South America; Central America; Caribbean Region; Argentina; Bolivia; Brazil; Chile; Colombia; Ecuador; French Guiana; Guyana; Paraguay; Peru; Suriname; Uruguay; Venezuela; Mexico; Belize; Costa Rica; El; Salvador; Guatemala; Honduras; Nicaragua; Panama; Aruba; Caribbean Netherlands; Curacao; Sint Maarten; West Indies	"Caribbean Region"[Mesh] "Central America"[Mesh] "Latin America"[Mesh] "South America"[Mesh] "Mexico"[Mesh]	'South and Central America'/exp 'Caribbean'/exp 'Mexico'/exp

Figure S1. Geographic distribution of the participants.

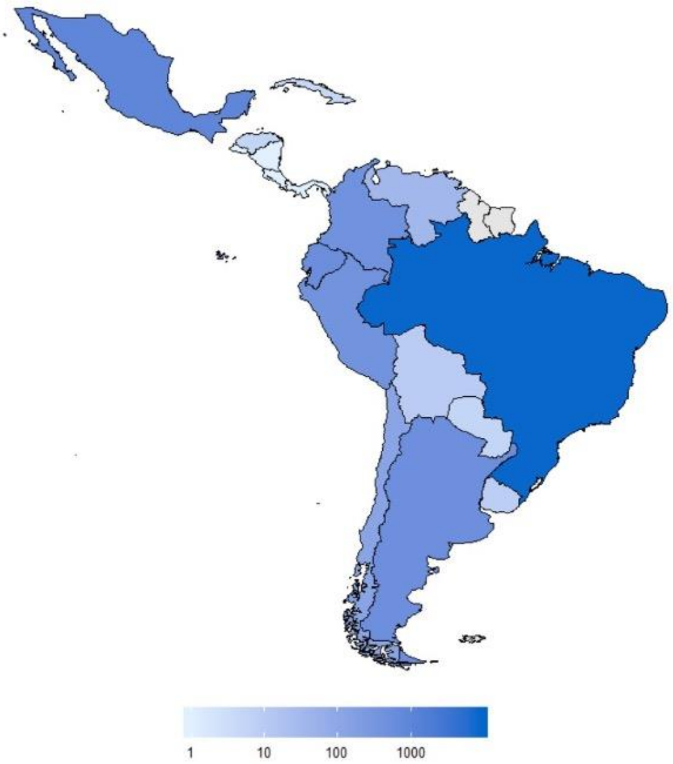

Supplement: Supplementary file 1 [file vaccines-13-00822-s001.zip › vaccines-3707552-supplementary.pdf]
